# 2-[(2,4,4,6,6-Penta­chloro-1,3,5,2λ^5^,4λ^5^,6λ^5^-triaza­triphosphinin-2-yl)aza­nid­yl]pyridinium

**DOI:** 10.1107/S160053681200013X

**Published:** 2012-01-07

**Authors:** Safaa A. Ahmed, Rosenani A. Haque, Zulikha H. Zetty, Hoong-Kun Fun, Wan-Sin Loh

**Affiliations:** a Department of Chemistry, College of Education Samarra, University of Tikrit, Tikrit 43001, Iraq; b School of Chemical Sciences, Universiti Sains Malaysia, 11800 USM, Penang, Malaysia; c X-ray Crystallography Unit, School of Physics, Universiti Sains Malaysia, 11800 USM, Penang, Malaysia

## Abstract

The title compound, C_5_H_5_Cl_5_N_5_P_3_, crystallizes as a zwitterion in which the pyridine N atom is protonated. An *S*(6) ring motif is formed *via* an intra­molecular C—H⋯N hydrogen bond. The triaza­triphosphinine ring adopts an envelope conformation, with one N atom displaced by 0.145 (1) Å from the other atoms. In the crystal, N—H⋯N and C—H⋯N hydrogen bonds link the mol­ecules into centrosymmetric dimers containing one *R*_2_^2^(7) ring motif and two *R*_2_^2^(8) ring motifs.

## Related literature

For background to the reactions of hexa­chloro­cyclo­triphosphazene, see: Polder & Wagner (1976 ▶). For a related structure, see: Coles *et al.* (2007 ▶). For ring conformations, see: Cremer & Pople (1975 ▶). For the stability of the temperature controller used in the data collection, see: Cosier & Glazer (1986 ▶).
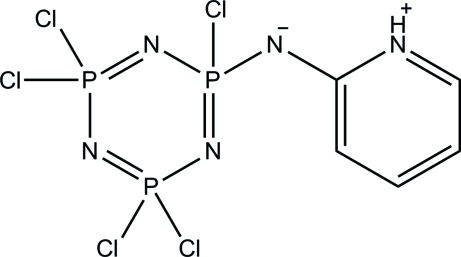


## Experimental

### 

#### Crystal data

C_5_H_5_Cl_5_N_5_P_3_
*M* *_r_* = 405.30
Monoclinic, 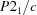

*a* = 8.8677 (1) Å
*b* = 14.7225 (2) Å
*c* = 12.3564 (2) Å
β = 119.355 (1)°
*V* = 1406.05 (3) Å^3^
*Z* = 4
Mo *K*α radiation
μ = 1.36 mm^−1^
*T* = 100 K
0.59 × 0.38 × 0.36 mm

#### Data collection

Bruker SMART APEXII CCD diffractometer
Absorption correction: multi-scan (*SADABS*; Bruker, 2009 ▶) *T*_min_ = 0.499, *T*_max_ = 0.640
19432 measured reflections
5116 independent reflections
4806 reflections with *I* > 2σ(*I*)
*R*_int_ = 0.017

#### Refinement

*R*[*F* ^2^ > 2σ(*F* ^2^)] = 0.023
*wR*(*F* ^2^) = 0.061
*S* = 1.09
5116 reflections
163 parameters
H-atom parameters constrained
Δρ_max_ = 0.54 e Å^−3^
Δρ_min_ = −0.39 e Å^−3^

### 

Data collection: *APEX2* (Bruker, 2009 ▶); cell refinement: *SAINT* (Bruker, 2009 ▶); data reduction: *SAINT*; program(s) used to solve structure: *SHELXTL* (Sheldrick, 2008 ▶); program(s) used to refine structure: *SHELXTL*; molecular graphics: *SHELXTL*; software used to prepare material for publication: *SHELXTL* and *PLATON* (Spek, 2009 ▶).

## Supplementary Material

Crystal structure: contains datablock(s) global, I. DOI: 10.1107/S160053681200013X/hb6587sup1.cif

Structure factors: contains datablock(s) I. DOI: 10.1107/S160053681200013X/hb6587Isup2.hkl

Supplementary material file. DOI: 10.1107/S160053681200013X/hb6587Isup3.cml

Additional supplementary materials:  crystallographic information; 3D view; checkCIF report

## Figures and Tables

**Table 1:** Hydrogen-bond geometry (Å, °)

*D*—H⋯*A*	*D*—H	H⋯*A*	*D*⋯*A*	*D*—H⋯*A*
N5—H1⋯N4^i^	0.84	2.16	2.9949 (14)	177
C2—H2*A*⋯N3	0.93	2.55	3.1538 (19)	123
C5—H5*A*⋯N1^i^	0.93	2.50	3.2220 (16)	135

Symmetry code: (i) 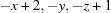.

**Fig. 1.**
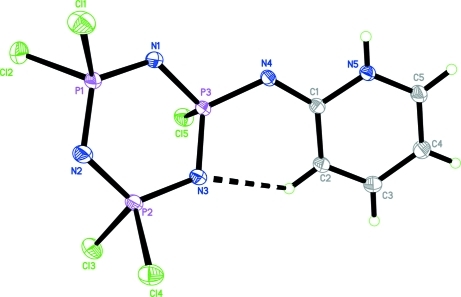
The molecular structure of the title compound, showing 50% probability displacement ellipsoids. The dashed line indicates the intramolecular hydrogen bond.

**Fig. 2.**
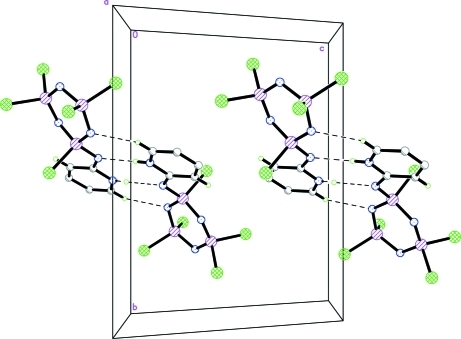
The crystal packing of the title compound, viewed along the a axis. H atoms not involved in the intermolecular interactions (dashed lines) have been omitted for clarity.

## supplementary crystallographic information

### Comment

Hexachlorocyclotriphosphazene is an inorganic six-membered cyclic compound consisting of alternating phosphorous and nitrogen atoms. It can also be considered as a trimer of azaphosphoryldichloride (NPCl_2_), which can be readily formed by the reaction of phosphorous pentachloride and ammonium chloride in chlorobenzene. The results of the reaction of phosphazene derivatives with nucleophile reagent strongly depend on reaction conditions whereas a series of various substitution derivatives can be formed (e.g. Polder & Wagner, 1976).

The title compound (Fig. 1), crystallizes as a zwitterion in which the pyridine N atom is protonated. An *S*(6) ring motif is formed *via* an intramolecular C2—H2A···N3 hydrogen bond (Table 1). The triazatriphosphinine ring (P1/N2/P2/N3/P3/N1) adopts an envelope conformation with the puckering parameters (Cremer & Pople, 1975), Q = 0.2087 Å; Θ = 138.0 (2)°; φ = 3.7 (4)° and it is comparable to a related stucture (Coles *et al.*, 2007). In the crystal (Fig. 2), N5—H1···N4 and C5—H5A···N1 hydrogen bonds (Table 1) link the molecules to form one *R*^2^_2_(7) ring motif and two *R*^2^_2_(8) ring motifs.

### Experimental

Hexachlorocyclotriphosphazene (0.5 g, 0.07 mol), 2-aminopyridine (0.26 g, 0.14 mol) and triethyl amine (0.14 g, 0.07 mol) were stirred in acetone at -80°C in liquid nitrogen bath for 5 h under anhydrous conditions. The obtained triethylammoniumchloride was filtered off under nitrogen and washed with fresh acetone and the solvent reduced to the minimum. Further, 10 ml of dried acetone was added the yield of the title product after deep freezing crystalization was about 60–66%. Colourless blocks were obtained by the slow evaporation of solvent at freezing temperature of acetone. *M.p.*: 455 K.

### Refinement

The N-bound hydrogen atom was located from the difference Fourier map and was fixed at their found positions with a riding model with *U*_iso_(H) = 1.2 *U*_eq_(N) [N–H= 0.8354 Å]. The remaining hydrogen atoms were positioned geometrically and were refined with a riding model with *U*_iso_(H) = 1.2 *U*_eq_(C) [C–H = 0.93 Å]. Four outliners were omitted for the final refinement, 3 16 4, 3 19 6, -3 16 7 and -3 19 9.

### Crystal data

**Table:**

C_5_H_5_Cl_5_N_5_P_3_	*F*(000) = 800
*M**_r_* = 405.30	*D*_x_ = 1.915 Mg m^−^^3^
Monoclinic, *P*2_1_/*c*	Mo *K*α radiation, λ = 0.71073 Å
Hall symbol: -P 2ybc	Cell parameters from 9860 reflections
*a* = 8.8677 (1) Å	θ = 2.4–32.6°
*b* = 14.7225 (2) Å	µ = 1.36 mm^−^^1^
*c* = 12.3564 (2) Å	*T* = 100 K
β = 119.355 (1)°	Block, colourless
*V* = 1406.05 (3) Å^3^	0.59 × 0.38 × 0.36 mm
*Z* = 4	

### Data collection

**Table:**

Bruker SMART APEXII CCD diffractometer	5116 independent reflections
Radiation source: fine-focus sealed tube	4806 reflections with *I* > 2σ(*I*)
graphite	*R*_int_ = 0.017
φ and ω scans	θ_max_ = 32.6°, θ_min_ = 2.3°
Absorption correction: multi-scan (*SADABS*; Bruker, 2009)	*h* = −11→13
*T*_min_ = 0.499, *T*_max_ = 0.640	*k* = −22→19
19432 measured reflections	*l* = −18→18

### Refinement

**Table:**

Refinement on *F*^2^	Primary atom site location: structure-invariant direct methods
Least-squares matrix: full	Secondary atom site location: difference Fourier map
*R*[*F*^2^ > 2σ(*F*^2^)] = 0.023	Hydrogen site location: inferred from neighbouring sites
*wR*(*F*^2^) = 0.061	H-atom parameters constrained
*S* = 1.09	*w* = 1/[σ^2^(*F*_o_^2^) + (0.0211*P*)^2^ + 1.0751*P*] where *P* = (*F*_o_^2^ + 2*F*_c_^2^)/3
5116 reflections	(Δ/σ)_max_ = 0.001
163 parameters	Δρ_max_ = 0.54 e Å^−^^3^
0 restraints	Δρ_min_ = −0.39 e Å^−^^3^

### Special details

**Table:**

Experimental. The crystal was placed in the cold stream of an Oxford Cryosystems Cobra open-flow nitrogen cryostat (Cosier & Glazer, 1986) operating at 100.0 (1) K.
Geometry. All e.s.d.'s (except the e.s.d. in the dihedral angle between two l.s. planes) are estimated using the full covariance matrix. The cell e.s.d.'s are taken into account individually in the estimation of e.s.d.'s in distances, angles and torsion angles; correlations between e.s.d.'s in cell parameters are only used when they are defined by crystal symmetry. An approximate (isotropic) treatment of cell e.s.d.'s is used for estimating e.s.d.'s involving l.s. planes.
Refinement. Refinement of *F*^2^ against ALL reflections. The weighted *R*-factor *wR* and goodness of fit *S* are based on *F*^2^, conventional *R*-factors *R* are based on *F*, with *F* set to zero for negative *F*^2^. The threshold expression of *F*^2^ > σ(*F*^2^) is used only for calculating *R*-factors(gt) *etc*. and is not relevant to the choice of reflections for refinement. *R*-factors based on *F*^2^ are statistically about twice as large as those based on *F*, and *R*- factors based on ALL data will be even larger.

### Fractional atomic coordinates and isotropic or equivalent isotropic displacement parameters (Å^2^)

**Table:**

	*x*	*y*	*z*	*U*_iso_*/*U*_eq_	
Cl1	1.37616 (5)	0.29414 (2)	0.46163 (3)	0.02552 (7)	
Cl2	1.44578 (4)	0.19325 (3)	0.27089 (3)	0.02419 (7)	
Cl3	0.89064 (4)	0.22782 (2)	−0.05598 (3)	0.02220 (7)	
Cl4	0.77111 (4)	0.35825 (2)	0.08438 (3)	0.02138 (6)	
Cl5	0.99461 (4)	−0.00326 (2)	0.13895 (3)	0.01986 (6)	
P1	1.25273 (4)	0.22143 (2)	0.30552 (3)	0.01580 (6)	
P2	0.92671 (4)	0.25229 (2)	0.11553 (3)	0.01506 (6)	
P3	0.99248 (4)	0.09616 (2)	0.25490 (3)	0.01336 (6)	
N1	1.18802 (13)	0.13083 (8)	0.33652 (9)	0.01774 (19)	
N2	1.12016 (15)	0.28591 (8)	0.19854 (10)	0.0216 (2)	
N3	0.85908 (13)	0.17106 (8)	0.16243 (9)	0.01651 (18)	
N4	0.93798 (13)	0.04813 (7)	0.34645 (9)	0.01398 (17)	
N5	0.75893 (13)	−0.03097 (7)	0.39730 (9)	0.01419 (17)	
H1	0.8457	−0.0360	0.4677	0.017*	
C1	0.78001 (15)	0.01276 (8)	0.30878 (10)	0.01275 (18)	
C2	0.63220 (16)	0.01682 (9)	0.18857 (10)	0.0171 (2)	
H2A	0.6394	0.0459	0.1244	0.021*	
C3	0.47895 (16)	−0.02188 (10)	0.16644 (11)	0.0200 (2)	
H3A	0.3833	−0.0190	0.0873	0.024*	
C4	0.46472 (16)	−0.06569 (10)	0.26157 (11)	0.0212 (2)	
H4A	0.3608	−0.0916	0.2469	0.025*	
C5	0.60793 (16)	−0.06919 (9)	0.37649 (11)	0.0190 (2)	
H5A	0.6018	−0.0981	0.4412	0.023*	

### Atomic displacement parameters (Å^2^)

**Table:**

	*U*^11^	*U*^22^	*U*^33^	*U*^12^	*U*^13^	*U*^23^
Cl1	0.02946 (16)	0.02404 (16)	0.01697 (12)	−0.00508 (12)	0.00667 (11)	−0.00396 (11)
Cl2	0.02044 (14)	0.03211 (18)	0.02312 (13)	−0.00176 (12)	0.01309 (11)	0.00253 (12)
Cl3	0.02719 (15)	0.02464 (15)	0.01649 (12)	−0.00050 (12)	0.01203 (11)	0.00132 (10)
Cl4	0.02328 (14)	0.01568 (13)	0.02180 (13)	0.00245 (10)	0.00843 (11)	0.00085 (10)
Cl5	0.02308 (14)	0.02248 (15)	0.01536 (11)	0.00251 (11)	0.01046 (10)	−0.00064 (10)
P1	0.01431 (13)	0.01830 (15)	0.01237 (12)	−0.00312 (11)	0.00467 (10)	0.00161 (10)
P2	0.01528 (13)	0.01473 (14)	0.01295 (12)	−0.00074 (10)	0.00522 (10)	0.00284 (10)
P3	0.01322 (12)	0.01484 (14)	0.01122 (11)	−0.00054 (10)	0.00537 (9)	0.00243 (9)
N1	0.0139 (4)	0.0200 (5)	0.0156 (4)	−0.0022 (4)	0.0043 (3)	0.0049 (4)
N2	0.0172 (5)	0.0210 (5)	0.0194 (4)	−0.0042 (4)	0.0035 (4)	0.0070 (4)
N3	0.0144 (4)	0.0170 (5)	0.0160 (4)	0.0001 (4)	0.0058 (3)	0.0054 (3)
N4	0.0145 (4)	0.0157 (4)	0.0117 (4)	−0.0018 (3)	0.0063 (3)	0.0015 (3)
N5	0.0137 (4)	0.0160 (5)	0.0116 (4)	−0.0016 (3)	0.0053 (3)	0.0008 (3)
C1	0.0149 (5)	0.0120 (5)	0.0117 (4)	0.0001 (4)	0.0067 (4)	−0.0003 (3)
C2	0.0157 (5)	0.0227 (6)	0.0115 (4)	−0.0004 (4)	0.0055 (4)	0.0017 (4)
C3	0.0157 (5)	0.0275 (6)	0.0135 (4)	−0.0016 (5)	0.0045 (4)	0.0007 (4)
C4	0.0156 (5)	0.0282 (7)	0.0172 (5)	−0.0055 (5)	0.0059 (4)	0.0005 (4)
C5	0.0172 (5)	0.0228 (6)	0.0162 (5)	−0.0041 (4)	0.0075 (4)	0.0028 (4)

### Geometric parameters (Å, °)

**Table:**

Cl1—P1	1.9987 (4)	N4—C1	1.3456 (15)
Cl2—P1	2.0011 (5)	N5—C5	1.3558 (16)
Cl3—P2	2.0146 (4)	N5—C1	1.3589 (14)
Cl4—P2	1.9912 (5)	N5—H1	0.8354
Cl5—P3	2.0548 (4)	C1—C2	1.4209 (15)
P1—N1	1.5719 (11)	C2—C3	1.3718 (18)
P1—N2	1.5834 (11)	C2—H2A	0.9300
P2—N3	1.5705 (11)	C3—C4	1.3996 (18)
P2—N2	1.5858 (11)	C3—H3A	0.9300
P3—N4	1.5967 (10)	C4—C5	1.3652 (17)
P3—N1	1.6031 (11)	C4—H4A	0.9300
P3—N3	1.6111 (11)	C5—H5A	0.9300
			
N1—P1—N2	120.13 (6)	P2—N3—P3	120.16 (7)
N1—P1—Cl1	108.18 (4)	C1—N4—P3	123.48 (8)
N2—P1—Cl1	108.41 (5)	C5—N5—C1	123.83 (10)
N1—P1—Cl2	109.20 (5)	C5—N5—H1	118.7
N2—P1—Cl2	107.98 (5)	C1—N5—H1	117.4
Cl1—P1—Cl2	101.32 (2)	N4—C1—N5	115.82 (10)
N3—P2—N2	119.12 (6)	N4—C1—C2	128.01 (10)
N3—P2—Cl4	108.31 (4)	N5—C1—C2	116.17 (10)
N2—P2—Cl4	107.93 (5)	C3—C2—C1	120.46 (11)
N3—P2—Cl3	111.07 (4)	C3—C2—H2A	119.8
N2—P2—Cl3	107.45 (5)	C1—C2—H2A	119.8
Cl4—P2—Cl3	101.487 (19)	C2—C3—C4	120.82 (11)
N4—P3—N1	107.76 (5)	C2—C3—H3A	119.6
N4—P3—N3	115.69 (6)	C4—C3—H3A	119.6
N1—P3—N3	114.83 (6)	C5—C4—C3	118.04 (12)
N4—P3—Cl5	106.73 (4)	C5—C4—H4A	121.0
N1—P3—Cl5	106.78 (5)	C3—C4—H4A	121.0
N3—P3—Cl5	104.34 (4)	N5—C5—C4	120.68 (11)
P1—N1—P3	121.89 (7)	N5—C5—H5A	119.7
P1—N2—P2	118.55 (7)	C4—C5—H5A	119.7
			
N2—P1—N1—P3	5.48 (12)	N1—P3—N3—P2	24.81 (10)
Cl1—P1—N1—P3	130.57 (7)	Cl5—P3—N3—P2	−91.71 (7)
Cl2—P1—N1—P3	−119.97 (7)	N1—P3—N4—C1	178.60 (10)
N4—P3—N1—P1	−144.81 (8)	N3—P3—N4—C1	48.55 (12)
N3—P3—N1—P1	−14.28 (11)	Cl5—P3—N4—C1	−67.03 (10)
Cl5—P3—N1—P1	100.84 (8)	P3—N4—C1—N5	175.77 (9)
N1—P1—N2—P2	−6.08 (12)	P3—N4—C1—C2	−5.33 (18)
Cl1—P1—N2—P2	−131.06 (7)	C5—N5—C1—N4	178.77 (12)
Cl2—P1—N2—P2	119.94 (7)	C5—N5—C1—C2	−0.27 (18)
N3—P2—N2—P1	16.53 (12)	N4—C1—C2—C3	−178.84 (13)
Cl4—P2—N2—P1	140.46 (7)	N5—C1—C2—C3	0.06 (18)
Cl3—P2—N2—P1	−110.81 (8)	C1—C2—C3—C4	0.3 (2)
N2—P2—N3—P3	−26.66 (11)	C2—C3—C4—C5	−0.4 (2)
Cl4—P2—N3—P3	−150.41 (6)	C1—N5—C5—C4	0.1 (2)
Cl3—P2—N3—P3	98.96 (7)	C3—C4—C5—N5	0.2 (2)
N4—P3—N3—P2	151.37 (7)		

### Hydrogen-bond geometry (Å, °)

**Table:**

*D*—H···*A*	*D*—H	H···*A*	*D*···*A*	*D*—H···*A*
N5—H1···N4^i^	0.84	2.16	2.9949 (14)	177
C2—H2A···N3	0.93	2.55	3.1538 (19)	123
C5—H5A···N1^i^	0.93	2.50	3.2220 (16)	135

Symmetry codes: (i) −*x*+2, −*y*, −*z*+1.
